# Role of Pyroptosis in Gynecological Oncology and Its Therapeutic Regulation

**DOI:** 10.3390/biom12070924

**Published:** 2022-07-01

**Authors:** Yi Huang, Ruiyun Li, Yuan Yang

**Affiliations:** 1 The First Clinical Medical College, Lanzhou University, Lanzhou 730000, China; huangy2020@lzu.edu.cn (Y.H.); liry21@lzu.edu.cn (R.L.); 2 The Reproductive Medicine Center, The 1st Hospital of Lanzhou University, Lanzhou 730000, China

**Keywords:** pyroptosis, cervical cancer, ovarian cancer, endometrial carcinoma

## Abstract

With the continuous advances in molecular biotechnology, many new cell death methods have been discovered. Pyroptosis is a programmed cell death process that differs from apoptosis and autophagy in cell morphology and function. Compared with apoptosis and autophagy, pyroptosis is primarily mediated by intracellular inflammasome and gasdermin D of the gasdermin protein family and involves the release of numerous inflammatory factors. Pyroptosis has been found to be involved in the occurrence and development of infectious diseases and other diseases involving the nervous system and the cardiovascular system. Recent studies have also reported the occurrence of pyroptosis in tumor cells. Accordingly, exploring its effect on tumors has become one of the research hotspots. Herein, recent research progress on pyroptosis is reviewed, especially its role in the development of gynecological tumors. As the pathogenesis of gynecological tumor is better understood, new targets have been introduced for the prevention and clinical treatment of gynecological tumors.

## 1. Introduction

With the continuous advances in molecular biotechnology, many new cell death methods have been discovered, including necroptosis, ferroptosis, and pyroptosis [1]. Pan-apoptosis is also a new concept proposed by Maridi in 2019 which emphasizes the interaction and coordination among the pathways involved in pyroptosis, apoptosis, and necrosis. Research in recent years has highlighted the interconnectedness among the pathways of these three processes [2]. However, gynecological oncology focuses on the unique procedures and biochemical mechanisms related to each process. Pyroptosis is a type of programmed cell death that is characterized by changes in osmotic pressure inside and outside the cell, swelling and rupture of the cell, release of inflammatory factors, lysosomes, and other cell contents outside the cell, and induction of inflammatory cascade reaction [3]. Pyroptosis occurs when the nucleus of cells remains intact, despite chromatin shrinkage and DNA damage. Inflammasomes associated with pyroptosis have been found in cancer cells [4,5,6]. These inflammasomes induce caspase-1, which leads to pyroptosis [7].

Currently, cancer is considered to be one of the main public health problems. A gynecological tumor occurs in female reproductive organs and is associated with a wide variety of clinical outcomes and different prevention and treatment methods. Unlike solid tumors of other organs, the outcomes of gynecological tumors are not only related to the patients, but also their offspring. Therefore, more severe challenges are associated with gynecological tumors. In the 1950s, some doctors successfully cured a patient with choriocarcinoma using methotrexate, ultimately beginning the era of solid tumor chemotherapy [8]. To date, gestational trophoblastic tumors are cured with chemotherapy alone, while other gynecological tumors are treated with chemotherapy as the main adjuvant therapy [9,10,11]. The advantage of chemotherapy is that it can guarantee the structural integrity of the organ. Furthermore, the damage to the ovary due to chemotherapy is relatively smaller than that by other therapies. As a result, chemotherapy is unique in protecting the endocrine and reproductive functions of the ovary [12]. Targeted drugs shift drug targets from cells to molecules, and targeted therapy has been one of the hallmarks of precision medicine [13]. Several targeted drugs have led to remarkable outcomes in the treatment of various solid tumors. However, in the case of gynecologic tumors, especially ovarian cancer, only a few targeted drugs with statistically significant survival benefits exist; thus, the reasons behind the lack of targeted drugs for gynecologic tumors are worth exploring.

Physiological cell death can control cell proliferation and inhibit tumor progression. Tumor cells can escape the recognition and attack of the body’s immune system in a variety of ways to survive and proliferate [14]. Cell death is the irreversible cessation of life and the end of life that often occurs in normal tissues and is necessary to maintain the function and morphology of tissues, including programmed death, apoptosis, and cell necrosis [15]. Apoptosis is a process strictly controlled by multiple genes. With the development of molecular biology techniques, the mechanisms of many types of cell apoptosis have been elucidated. An imbalance in apoptosis may be directly or indirectly related to the occurrence of many diseases [16]. In recent years, many studies have sought to explore the molecular mechanism of pyroptosis in the formation and development of multiple tumors [17,18,19]. Pyroptosis is related to the progression of various diseases, especially the progression of human malignant tumors [20]. In 1996, pyroptosis was first reported in bacteria and is believed to be a form of gasdermin (GSDM)-mediated cell death [21]. Notably, pyroptosis may be an important natural immune response of the body, which plays an important role in the fight against infections [22]. Previously, a report published by Bergsbaken et al. revealed that pyroptosis can affect the clearance of pathogens, thereby reducing the adaptive immune response and causing extensive tissue damage [23]. In this review, the pathogenesis, tumor progression, and therapeutic strategies of pyroptosis in gynecological malignant tumors were systematically described, providing new ideas for the treatment of gynecological tumors.

## 2. Mechanism of Pyroptosis

In 1996, Cheng et al. [21] found that mouse macrophages could undergo apoptosis accompanied by the release of interleukin-1β (IL-1β) after being infected with *Shigella* spp., and subsequent studies revealed that this was a new type of programmed cell death which is independent of caspase-3 activity and is related to caspase-1 activity [24]. Although *Shigella*-induced death can still occur in caspase-3-deficient macrophages, it is inhibited by caspase-1 specific inhibitor or in caspase-1 gene knockout [25]. In 2000, Brennan and Cookson first described this particular type of programmed cell death as pyroptosis [26]. In 2006, Miao et al. [27] verified the above findings, confirming that caspase-1-dependent programmed cell death is accompanied by the release of numerous pro-inflammatory factors. Pyroptosis is a type of programmed cell death that activates inflammasomes [28]; this inflammatory cell death is harmful to the body, and the resulting disease is associated with chronic and continuous progress. Table 1 summarizes the difference between pyroptosis and other types of cell death.

GSDMs are expressed in a variety of cell types and tissues and belong to a family of proteins with multiple functions [29]. At present, six GSDM proteins have been identified in humans. Based on the crystal structure of mouse GSDM A (GSDMA), mouse GSDM D (GSDMD), and human GSDMD, it has been discovered that all GSDMs share the same self-inhibition mechanism. In fact, GSDM C-terminal domain (GSDM-CTD) can inhibit the oligomerization of GSDM N-terminal domain (GSDM-NTD) and thus inhibit its membrane perforation function [30,31,32].

In humans, GSDMA is mainly expressed in the digestive tract, breast, and skin tissues. T cells also express GSDMA [33]. GSDMA is similar to other GSDMs, its N-terminal domain is responsible for the membrane perforation, and its C-terminal and N-terminal domains can form self-inhibited structures under physiological conditions [34]. The N-terminal domain of GSDMA can also interact with the tumor necrosis factor receptor-associated protein 1 (TRAP1) present on the mitochondria; TRAP1 regulates the permeability of the mitochondrial membrane. Since GSDMA can inhibit the function of TRAP1, GSDMA can damage cellular and mitochondrial membranes [35], thereby triggering the accumulation of intracellular reactive oxygen species (ROS), which initiates apoptosis through the mitochondrial pathway. To date, proteins with GSDMA cleavage ability and cytokines specifically released in GSDMA-induced pyroptosis have not been reported.

GSDM B (GSDMB) is mainly expressed in the human airway epithelium, esophagus, stomach, liver, small intestine, colon, and other tissues. Many alternative splicing variants of GSDMB have been detected in humans, and one transcript (encoded by exon 6) contains a caspase-1 cleavage site [36]. Studies have shown that caspase-1 cleaves this subtype and induces pyroptosis [37,38]. GSDMB releases its N-terminal domain through the hydrolysis of Lys229/Lys223, resulting in the pyroptosis of target cells [36].

GSDM C (GSDMC) is mainly expressed in the spleen, trachea, stomach, and digestive tract [39]. GSDMC is similar to other GSDMs, as its N-terminal domain can form holes in the cell membrane [40]. According to recent literature, the phosphorylated signal transducer and activator of transcription 3 (STAT3) and programmed cell death receptor ligand 1 (PDL1) complex can promote the expression of GSDMC in tumor cells [41]. In the presence of tumor necrosis factor-alpha (TNF-α), caspase-8 is activated in tumor cells. Activated caspase-8 cleaves GSDMC at D365, leading to the release of its N-terminal domain, which transforms cell apoptosis into tumor focal death [42]. A leucine zipper is located in the C-terminal domain of GSDMC that may recognize specific DNA sequences [43].

**Figure 1:**
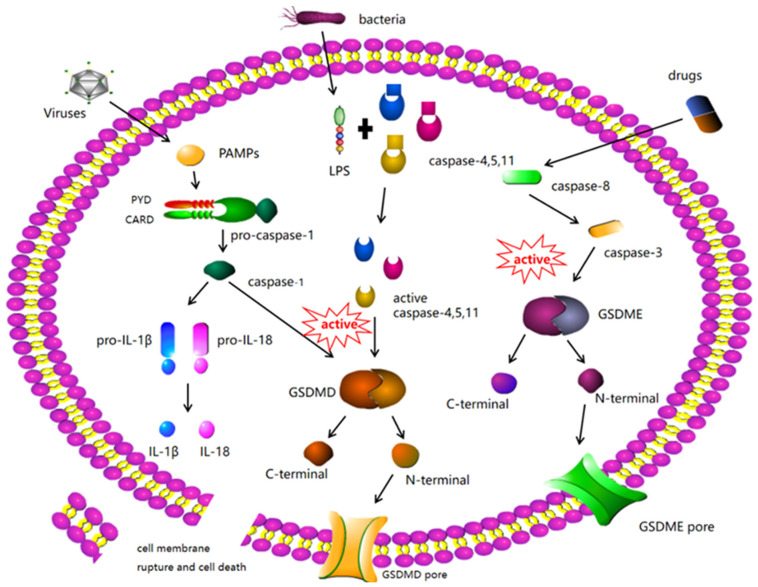
Molecular mechanisms of pyroptosis-regulated cell death. The stimulated inflammasome components trigger the cleavage of Caspase-1. Then, Caspase-1 can significantly cleave GSDMD to form GSDMD N-fragment and plasma membrane pores formation, leading to pyroptosis-regulated cell death. Stimulated Caspase-1 leads to the maturation and secretion of IL-1β and IL-18 inflammatory factors. Besides, LPS can bind to the Caspase-4/5/11 precursor, leading to pyroptosis-regulated cell death. Caspase-3/GSDME can also lead to pyroptosis-regulated cell death. Notably, caspase-8 triggers Caspase-3. Then, the stimulated Caspase-3 cleaves GSDME to form GSDME N-fragments, leading to plasma membrane pores formation, cell swelling, and pyroptosis.

GSDMD is widely expressed in a variety of tissues and leukocytes [44]. GSDMD was the first executor to be identified in the process of pyroptosis [45]. According to current studies, GSDMD contains the cleavage sites of caspase-1 (human), caspase-11 (mouse), caspase-4, and caspase-5 [46]. Caspase-4, -5, and -11 are involved in non-classical inflammatory body signaling pathways that can be activated by viruses or lipopolysaccharide (LPS) [47]. After activation, caspases-4, 5, and 11 cleave GSDMD and release its N-terminal domain to induce pyroptosis. To date, no cytokines specifically released in caspase-11-GSDMD-mediated pyroptosis have been identified. Caspase-1-GSDMD-mediated pyroptosis is the most classical model of pyroptosis [48]. Caspase-1 belongs to the classic inflammasome signaling pathway. Pathogen-associated molecular patterns (PAMPs), abnormal cell homeostasis, and a variety of extracellular danger signals can activate caspase-1 [49,50]. Activated caspase-1 cleaves GSDMD to release its N-terminal domain to induce pyroptosis and cleaves IL-1β and interleukin-18 (IL-18) precursors for maturation [51]. Caspase-1-GSDMD-mediated pyroptosis is thus often accompanied by the release of activated IL-Iβ and IL-18 (Figure 1).

GSDM E (GSDME) was initially identified to be encoded by a gene responsible for autosomal dominant non-syndromic hearing loss diseases, but was later included in the GSDM protein family due to its sequence and structural similarity with that of the GSDM family proteins [52]. GSDME is expressed in a variety of human tissues, including the brain, endometrium, placenta, and intestine. Caspase-3 can activate GSDME through direct or indirect cleavage and initiate pyroptosis [53]. Caspase-3 cleaves interleukin-16 (IL-16) as well as GSDME [54]. IL-16 is similar to IL-Iβ and IL-18 because of the following reasons: it is a proinflammatory cytokine, it needs to be cleaved and activated to perform its biological function, and it also has no signaling peptides and cannot be released from the cell by the classic cytokine release process. Scientists have hypothesized that activated IL-16 might be a cytokine specifically released in GSDME-induced pyroptosis based on a comparison of the molecular mechanism of GSDME-IL-16 with that of GSDMD-IL-1β and -IL-18 [55].

## 3. Pathways Associated with Pyroptosis

There are classical caspase-1 dependent pathways and non-classical caspase-4/-5/-11 dependent pathways. In the classical pyroptosis pathway, pro-caspase-1 contain caspase recruitment domain (CARD), apoptosis-associated speck-like protein (ASC), and nod-like receptor 1(NLRP1). Nod-like receptor protein 3(NLRP3), NLR family CARD domain-containing protein 4 (NLRC4), and absent in melanoma 2(AIM2) were indirectly linked to pyrin and other proteins [56]. Formation of the inflammasome, activation cleavage of pro-caspase-1, and formation of active cleaved caspase-1 promote membrane perforation and death. Further, cleaved caspase-1 cleaves IL-1β and IL-18 precursors, promoting IL-1β and IL-18 production and their release into the extracellular environment, thus amplifying the inflammatory response [57,58,59]. In the non-classical pyroptosis pathway, bacterial LPS and caspase-4/-5/-11 activate GSDMD and mediate cell membrane lysis and pyroptosis, eventually leading to the production of IL-1β [47].

The inflammasome is an intracellular multiprotein complex defined by its core protein pattern-recognition receptor (PRR). The main PRRs of inflammatory bodies are the nod-like receptor (NLR) family, ASC containing CARD, and pro-caspase-1. NLR is mainly composed of three domains: the end-effect binding domain of N can be divided into caspase recruitment domain, pyrin domain, or baculovirus apoptosis inhibition repeat domain; the center has a nucleotide-binding oligomerization domain; and the C-terminal consists of leucine-rich repeats. After recognizing endogenous or exogenous danger signals, NLR can recruit pro-caspase-1 by promoting CARD interaction or recruit CARD-containing adaptor protein ASC through pyrin domain. Further, ASC acts as a bridge to pro-caspase-1, thereby activating caspase-1 [60,61]. Mature caspase-1 cleaves pro-IL-1β and pro-IL-18 to further induce the maturation of IL-1β and IL-18. In general, cytokines, such as IL-1β and IL-18 have multiple biological functions and play a central role in immune response [62]. The assembly of NLRP3, AIM2, NLPR1, and NLRC4 inflammasome is strictly dependent on bridge ASC, NLRP1, and NLRC4, which have CARD domains that directly recruit caspase-1. Therefore, NLRP1 and NLRC4 can be assembled as a part of ASC, which is activated by canonical inflammatory bodies. In addition, caspase-11 and caspase-8 promote the activation of the non-classical inflammasome, and caspase-11 plays an important role in the activation of caspase-1 and caspase-3 [63].

In fact, Caspase 3 and Caspase 8 are major players in apoptosis. However, with progress in research, caspase-3 and/or caspase-8 have been found to induce pyroptosis. Caspase-3 is an important terminal shearing enzyme that can rapidly recognize and cleave GSDMD and form GSDMD-N, which promotes the formation of cell membrane pores and leads to pyroptosis [64]. Caspase-8 is involved in signaling pathways that regulate key molecules and can lyse GSDMC and GSDME to promote pyroptosis [65]. TGF-β-activated kinase-1 (TAK1) is a basic factor regulating NF-κB signal transduction. Evidence suggests that the inhibition of TAK1 by Yersinia exo-protein J (YOP-J) or other less critical inhibitory molecules promotes caspase-8-mediated cleavage of GSDMD and forms cell membrane pores, leading to pyroptosis [2].

## 4. Pyroptosis and Tumor

The pathogenesis of tumors is relatively complex. However, inflammasomes have also been found in tumor cells; these inflammasomes can promote and inhibit tumor growth [66]. As the inflammasome is a key molecule that induces caspase-1 to undergo pyroptosis, it may be an important node in the association between tumor cells and pyroptosis. Different tumors involve different inflammasomes. For example, NLRP3 is widely present in tumor cells and is associated with nasopharyngeal cancer, colorectal cancer, and lung adenocarcinoma [5]. In addition, liver cancer is associated with the AIM2 inflammasome [67]. Although pyroptosis can be presumed to be related to tumors, the relationship between pyroptosis and tumor is complicated. Pyroptosis can not only inhibit the occurrence and development of tumors, but also promote inflammatory cell death. Pyroptosis can also promote tumor growth by forming a suitable microenvironment for tumor cell growth [68]. In the tumor immune microenvironment, tumor cells are protected by mesenchymal cells, extranuclear cells, cytokines, chemokines, and metabolites so that tumor cells can survive. Pyroptosis can inhibit and promote tumor cells in the tumor immune microenvironment. It can reprogram tumor immune microenvironment into immune stimulation state through damage-associated molecular patterns (DAMPs) released after osmotic lysis, thus inhibiting tumor cell growth and metastasis. It can also promote the growth of tumor cells under the action of inflammatory factors. Pyrotopia may have specific effects on tumor immune microenvironment and promote immune surveillance. Thus, pyroptosis is a potential new target for cancer treatment.

Many molecules can induce pyroptosis of tumor cells, and their effects are not consistent in different tumors. Chemotherapeutic drugs have always played an important role in the treatment of tumors, especially extensive malignant tumors. Different types of chemotherapeutic drugs have different targets. Owing to a deeper understanding of pyroptosis, some chemotherapeutic drugs have been proven to induce cell pyroptosis. Some anticancer drugs that affect cancer by inducing different pyroptosis pathways are described in Table 2.

In 2017, a study published in Nature revealed that topotecan, irinotecan, etoposide, and cisplatin can induce pyroptosis in Jurkat cells and Me Wo cells; adriamycin and fluorouracil can induce pyroptosis in HeLa cells; and adriamycin, actinomycin D, or topotecan can induce pyroptosis in NCI-H522 cells [78]. Notably, these pathways are induced by caspase-3 and GSDMD, and the widespread presence of GSDMD can lead to pyroptosis of normal cells during chemotherapy. Other studies have reported that paclitaxel and cisplatin can cause pyroptosis of lung cancer A549 cells, and cisplatin has a stronger pyroptosis ability than paclitaxel. Paclitaxel can also induce pyroptosis in nasopharyngeal carcinoma cells [71]. Loplatin has been confirmed to induce pyroptosis of HT-29 and HCT116 cells in the treatment of colon cancer [54]. Previously, chemotherapy drugs were believed to induce apoptosis of tumor cells; however, owing to a deeper understanding of pyroptosis, a process that was initially thought to be a part of apoptosis was later proven to be a part of pyroptosis.

Some studies have shown that the cell membrane of gastric cancer cells is swollen and broken after 5-FU treatment. Further, GSDME was revealed to transform caspase-3-dependent apoptosis induced by anti-cancer drugs into pyroptosis of gastric cancer cells, which is helpful for re-understanding the effects of chemotherapy drugs [79]. However, whether combination therapy can improve patient outcomes is controversial. Pyroptosis has also been discussed in molecular studies, which suggests that it can be used to enhance the efficacy of chemotherapy drugs. For example, the polo-like kinase 1 (PLK1) inhibitor, BI2536, enhances the chemical sensitivity of cisplatin by inducing pyroptosis in esophageal squamous cell carcinoma, and cisplatin combined with BI2536 can induce pyroptosis in esophageal squamous cell carcinoma at low doses [80]. Eukaryotic plant factor 2 kinase (eEF-2K) plays a synergistic role with doxorubicin in the treatment of melanoma cells. Further, the inhibition of eEF-2K has been observed to inhibit pyroptosis of tumor cells [81]. eEF-2K indirectly enhances the antitumor effect of doxorubicin.

## 5. Gynecological Oncology

Gynecological tumors occur in female reproductive organs, with a wide variety of clinical outcomes and different prevention and treatment methods. Unlike solid tumors of other organs, the outcomes of gynecological tumors are not only related to the patients, but also their offspring. Therefore, more severe challenges are associated with gynecological tumors. Molecular targeted therapy has changed the treatment strategy and concept of tumors. Further, individualized and precise treatment of gynecological tumors has improved the prognosis and quality of life of patients. Research and screening of more efficient, accurate, and safe targeted drugs are the current focal points of gynecological tumor research. In recent years, the significance of pyroptosis in the treatment of gynecological tumors has been widely investigated, and many new achievements have been made. Pyroptosis can be a new target for the treatment of gynecological tumors [76]. A summary of studies on the role of pyroptosis and related compounds in gynecological oncology is provided in Table 3.

## 6. Pyroptosis and Endometrial Carcinoma

Endometrial carcinoma is a group of epithelial malignant tumors occurring in the endometrium, most commonly in perimenopausal and postmenopausal women. Recently, the incidence of this tumor has been increasing, with approximately 58,500 new cases and 7000 deaths worldwide each year [93]. A close relationship exists between inflammation and tumor. Several studies have found that inflammation is rich in inflammatory cells and cytokines. The inflammatory microenvironment of chemokines and DNA damage-promoting substances can lead to the occurrence, development, and metastasis of tumors. Endogenous and exogenous estrogen, genetic factors, and oncogenic mutations play a role in the development of endometrial carcinoma. Periodic changes in the endometrium are also related to chronic inflammation. Liu et al. [94] revealed that the expression of the inflammasome NLRP3, which is associated with pyroptosis, was significantly increased in endometrial cancer, and the increase was positively correlated with clinical and pathological stages of the cancer. Yang et al. [82] confirmed that hydrogen can induce the GSDMD pathway in endometrial cancer to mediate pyroptosis, thereby affecting the biological behavior of endometrial cancer cells. Chang et al. [95] found that in patients with endometrial cancer, the expression levels of pyroptosis-related inflammasomes AIM2 and NLRP3 were significantly higher than those in the normal group. The expression levels of NLRP3, GSDMD, caspase-1, and IL-1β were also significantly higher in atypical hyperplasia and carcinoma tissues than in benign endometrial tissues. Overactivated inflammasome and pyroptosis-related proteins in patients with endometrial cancer induce pyroptosis, inhibit the progression of endometrial cancer, and provide specific targets for clinical treatment.

## 7. Pyroptosis and Cervical Cancer

The incidence and mortality of cervical cancer is fourth among female malignant tumors worldwide and second in developing countries [96]. Persistent infection of human papillomavirus 16 (HPV16) and human papillomavirus 18 (HPV18) is the main cause of cervical cancer. In HPV-infected cells, the inflammasome AIM2 acts as a tumor suppressor by activating caspase-1 to promote pyroptosis of tumor cells [86]. The caspase-3/GSDME axis is involved in loplatin-mediated pyroptosis of cervical cancer cells. These findings suggest that GSDME-mediated pyroptosis is a novel mechanism to kill tumor cells, and the caspase-3/GSDME pathway provides a new approach for tumor chemotherapy [87]. HPV E7 recruits the estriol (E_3_) ligase recombinant protein of human tripartite motif-containing 21 (TRIM21) to ubiquitinate and degrade IL-16 inflammasome, leading to the inhibition of pyroptosis and self-evasion of immune surveillance. The upregulation of Mir-214 in cervical cancer patients and cervical cancer cell lines can promote pyroptosis of cervical cancer cells by enhancing NLRP3 expression [84]. Tong et al. [85] found that tanshinone II A could promote pyroptosis and inhibit tumor cell proliferation by regulating the Mir-145/GSDMD signaling pathway. After treatment with tanshinone II A, the expression levels of Mir-145, GSDMD, IL-18, and IL-1β, proteins related to pyroptosis, were found to be increased in Hela cells. Pyroptosis depends on the pore-forming activity of the GSDM protein family and inhibits the development of cervical cancer through the activity of caspase-1 and inflammatory factors.

## 8. Pyroptosis and Ovarian Cancer

Ovarian cancer is one of the most common cancers in women, with a five-year survival rate of less than 45% [97]. Studies have shown that the development of ovarian cancer can be inhibited by inducing pyroptosis. Further, long non-coding RNA growth arresting-specific transcript 5 (LNC-RNA-GAS5) can activate apoptosis-related blotk-like protein, caspase-1, and IL-1β. Caspase-1 has been demonstrated to activate GSDMD to form the GSDMD-N terminal fragment. GSDMD-N specifically binds to the cell membrane and oligomerizes to form membrane pores, causing cell swelling and pyroptosis, which inhibits the proliferation of ovarian cancer cells [90]. According to Qiao et al. [88], α-Neta inhibited the proliferation of epithelial ovarian cancer cells by directly inducing pyroptosis via the activation of caspase-4 cleavage of GSDMD. With further elucidation of the mechanisms involved in pyroptosis, pyroptosis can be considered as an efficient target for the diagnosis and treatment of ovarian cancer. Nobilin reduces mitochondrial membrane potential and induces the generation of reactive oxygen species and autophagy of human ovarian cancer cells (HOCCs), leading to GSDMD/GSDME-mediated pyroptosis [89].

## 9. Prospects and Summary

In conclusion, pyroptosis is a pro-inflammatory programmed cell death process that relies on the pore-forming activity of the GSDM protein family and plays a role in the occurrence and development of gynecological tumors through the activation of the caspase proteins and inflammatory factors. However, the specific pathways and mechanisms associated with pyroptosis in gynecologic tumors have not been fully elucidated. Current studies have shown that the inflammasome is closely related to gynecological malignant tumors and causes thermal death of tumor cells, which can be used as a feasible therapeutic target for gynecological tumors. Therefore, the regulation of GSDMs, key effectors of pyroptosis, is a new strategy and effective approach for the treatment of gynecological tumors. For example, certain drugs or molecules (metformin, anthocyanin, DHA(Docosahexaenoic Acid), 2-(naphthoyl) ethyl trimethylammonium iodide) can promote pyroptosis of cancer cells by activating GSDMD. Most tumor cells inhibit GSDME via methylation modification with a DNA methyltransferase inhibitor. Decitabine can promote the expression of GSDME and induce pyroptosis of tumor cells. Further, the increase in intracellular reactive oxygen species can stimulate caspase-3 to cut off GSDME and induce pyroptosis of tumor cells. The occurrence of pyroptosis also increases the sensitivity of tumor cells to chemotherapeutic drugs; thus, the combination of chemotherapeutic drugs and GSDM protein activator may be beneficial for tumor treatment. The braftovi/mektovi (BRAF/MEK) inhibitor-induced pyroptosis can induce changes in the tumor immune microenvironment and improve the therapeutic effect of drug-resistant melanoma, which provides a new direction for scorch-mediated anti-tumor immunity and treatment of drug-resistant tumors. Although some members of the GSDM protein family are known to induce pyroptosis and participate in the treatment of tumors, how they precisely regulate its expression level and promote tumor cell death are unknown. Further, avoiding the generation of a tumor-friendly microenvironment remains key in future cancer therapy. The inflammatory response caused by pyroptosis can cause strong tissue damage. Thus, how to kill tumor cells and avoid an inflammatory cytokine storm are current challenges in the application of heat death.

Pyroptosis is a newly discovered way of cell death in recent years, and its regulatory mechanism needs to be further explored. LncRNAs (long noncoding RNA) are RNA transcripts over 200 nucleotides in length that are generally considered incapable of encoding amino acids and are involved in many physiological and pathological processes. Stimulated by exogenous and endogenous factors, abnormal expression of lncRNAs affects the expression of pyroptosis pathway-related proteins through two indirect ways: miRNAs and downstream proteins/pathways, and they directly regulate the inflammasome, which plays an important role in pyroptosis, thus regulating the pathological process of various diseases. In addition, lncRNAs often act as endogenous sponges for miRNAs, affecting the levels of their targeted proteins, including those associated with the pyroptosis pathway [98]. Accumulating evidence suggests that lncRNAs play important roles in processes such as inflammation, oxidative stress, and pyroptosis. LncRNA can increase the expression of NLRP3, and up-regulation of lncRNA expression can activate the NLRP3, trigger tumor pyroptosis, and lead to cell death. With more research, more lncRNAs related to pyroptosis will be discovered, and the regulatory mechanisms of their pyroptotic pathways will become clearer [99]. Therefore, altering lncRNAs in vivo through novel therapies may be a promising approach for the diagnosis and treatment of pyroptosis-related diseases.

More and more studies have focused on pyroptosis in tumors. Currently, the research mainly focuses on compounds or molecules that activate inflammasome such as NLRP3 and AIM2 and promote pyroptosis, which are promising as new drugs for the treatment of tumors. However, there are still many pending issues in the study of pyroptosis. For example, cell death may coexist in various ways in the state of disease, and activation of inflammasome and caspase and other proteins may not all cause pyroptosis. How to accurately distinguish and identify different types of cell death? This requires the development of new identification methods. In addition, as a key pyroptosis executor, there are few studies on the molecular regulatory mechanism of GSDMD itself. Besides upstream inflammasome and caspase protein, whether there are other effector targets remains to be discovered. Owing to differences in the expression and activity of pyroptosis in different gynecological diseases, its role is also different. Therefore, the application of pyroptosis in the pathogenesis, diagnosis, and treatment of gynecological tumors requires further comprehensive research.

## Figures and Tables

**Table 1:** Summary of studies on the difference between pyroptosis and other types of cell death.

	Pyroptosis	Apoptosis	Necrocytosis	Autophagy	Ferroptosis
Death form	Programmed	Programmed	Programmed	Programmed	Programmed
induction	Pathological stimulus	Gene regulation	Physical and chemical stimulation or pathogen infection	Pathological irritation or nutritional deficiency	Excessive lipid peroxidation
cytomembrane	Cell pore formation	invagination	cleavage	integrity	integrity
cell nucleus	integrity	pyknosis and fragmentation	pyknosis and fragmentation	Fusion with lysosomes	integrity
organelle	deformation	integrity	deformation	Autophagosome phagocytosis	deformation
DNA	Random degradation	Ladder banded degradation	Random degradation	Random degradation	Random degradation
cell morphology	Ballooning degeneration	Cell shrinkage	cellular swelling	Crescent or cup shaped	cellular swelling
Key molecules	caspase-1/3/4/5/8/11, IL-18, IL-1β, GSDMD	Caspase-2/3/6/7/8/9/10,P53, Bcl-2, AOP-1, TNF, CHOP, JNK	RIP1, RIP3, MLKL, TNF-α, TNFR1, FLIP,	mTOR, RAS, MAPK, ERK, Bcl-2	RIP1, RIP3,MLKL, PKC, MAPK, AP-1, ROS

**Table 2:** Drugs inducing pyroptosis signaling pathways for regulating and treating cancers.

Cancer Types	Therapeutic Drugs	Mechanisms of Pyroptosis Induction	References
Lung cancer	Polyphyllin VI	ROS/NF-κB/NLRP3/GSDMD	[69]
	Cisplatin and paclitaxel	Caspase-3/GSDME	[68]
Colorectal cancer	Arsenic trioxide + Ascorbic acid	ROS/Caspase-1/IL-1β,IL-18	[70]
	Lobaplatin	Caspase- 3/GSDME	[54]
Gastric cancer	5-fluorouracil, cisplatin	Caspase-3/GSDME	[71]
Neuroblastoma	Dasatinib	Caspase-3/GSDME	[72]
Melanoma	Iron + CCCP	Caspase-3/GSDME	[73]
Breast cancer	Cisplatin	MEG3/NLRP3/caspase-1/GSDMD	[74]
Nasopharyngeal carcinoma	Taxol	Caspase-1/GSDMD	[75]
Skin cancer	Doxorubicin	Caspase-3/GSDME/eEF-2K	[76]
Esophageal cancer	Metformin	miR-497/PELP1/Caspase1/GSDMD	[77]

**Table 3:** Summary of studies on the role of pyroptosis and related compounds in gynecological oncology.

Gynecological Oncology	Interventions	Mechanism of Action	Application	In Vitro/In Vivo	Animal Model	References
endometrial cancer	Hydrogen	ROS/NLRP3/caspase-1/ GSDMD	Suppression	Both	female SPF grade BALB/c-nude mice	[82]
cervical cancer	MiRNA-214	NLRP3	Suppression	Both	wistar female rats	[83]
	HPV E7	inhibited the cleavage of GSDMD	Promotion	In vitro		[84]
	tanshinone II	regulating miR-145/ GSDMD	Suppression	In vitro		[85]
	SIRT1	eliminate AIM2	Promotion	Both	wistar female rats	[86]
	Lobaplatin	caspase-3/GSDME	Suppression	In vitro		[87]
ovarian cancer	alpha-NETA	GSDMD/caspase-4	Suppression	Both	female SPF grade BALB/c-nude mice	[88]
	Nobiletin	increased cleavage levels of GSDMD and GSDME	Suppression	In vitro		[89]
	lncRNA GAS5	inhibits inflammasome formation and pyroptosis	Suppression	Both	female SPF grade BALB/c-nude mice	[90]
	LncRNA HOTTIP	nhibits cell pyroptosis by targetingmiR-148a-3p/AKT2 axis	Suppression	In vitro		[91]
	Osthole	GSDME	Suppression	In vitro		[92]

“Suppression” indicates that the intervention suppresses cancers. “Promotion” indicates that the intervention promotes cancers. “In vitro/in vivo” indicates whether the study was performed in vivo, in vitro, or both.

## Data Availability

Not applicable.

## Abbreviations

NLRP3	nod-like receptor protein 3
AIM2	absent in melanoma 2
IL-1β	interleukin-1β
GSDMs	Gasdermins
GSDMA	GasderminA;GSDMB:Gasderminb
GSDMC	GasderminC
GSDMD	GasderminD
GSDME	GasderminE
DFNB59	autosomal recessive deafness-59
GSDMs-CTD	Gasdermins C-terminal domain
GSDMS-NTD	Gasdermins D-terminal domain
TRAP1	tumor necrosis factor receptor-associated protein 1
ROS	reactive oxygen species
CTLs	cytotoxic T lymphocytes
STAT3	signal transducer and activator of transcription 3
PDL1	programmed cell death receptor ligand 1
TNF-α	tumor necrosis factor alpha
IL-18	interleukin-18
IL-16	interleukin-16
CARD	caspase recruitment domain
ASC	apoptosis-associated speck-like protein
NLRP1	nod-like receptor 1
NLRP3	nod-like receptor protein 3
NLRC4	NLR Family CARD domain-containing protein 4
LPS	lipopolysaccharide
PRR	protein pattern-recognition receptor
NLR	nod-like receptor
APAF-1	apoptotic peptidase activating factor-1
TAK1	TGF-β-activated kinase-1
YOP-J	Yersinia exo-protein J
PLK1	polo-like kinase 1
eEF-2K	Eukaryotic plants factor 2 kinase
HPV16	human papillomavirus 16
HPV18	human papillomavirus 18
TRIM21	recombinant protein of human tripartite motif-containing 21
LNC-RNA-GAS5	long non-coding RNA growth arresting-specific transcript 5
HOCCs	human ovarian cancer cells
BRAF/MEK	braftovi/mektovi
LncRNAs	long noncoding RNA.
